# Serum 8-hydroxy-2-deoxyguanosine as a marker of DNA oxidative damage in horses with recurrent airway obstruction

**DOI:** 10.1186/s13028-016-0215-6

**Published:** 2016-06-07

**Authors:** Artur Niedzwiedz, Hieronim Borowicz, Lidia Januszewska, Iwona Markiewicz-Gorka, Zbigniew Jaworski

**Affiliations:** 1 Department of Internal Diseases with Clinic for Horses, Dogs and Cats, Wroclaw University of Environmental and Life Sciences, pl. Grunwaldzki 47, 50-366 Wrocław, Poland; 2 Department of Hygiene, Wroclaw Medical University, J. Mikulicza-Radeckiego 7, 50-435 Wrocław, Poland; 3 Department of Horse Breeding and Riding, University of Warmia and Mazury, Prawocheńskiego 2, 10-720 Olsztyn, Poland

**Keywords:** Heaves, Horse, DNA damage, Oxidative damage, RAO, Oxidative stress

## Abstract

**Background:**

It has been reported that equine recurrent airway obstruction (RAO) is a state of oxidative stress. Oxidant-antioxidant imbalance is known to increase the conversion of deoxyguanosine to 8-hydroxy-2-deoxyguanosine (8-OHdG) in DNA. 8-OHdG can easily be measured using ELISA tests in serum or urine samples. In this study, we analysed serum 8-OHdG levels in horses with recurrent airway obstruction and in healthy controls.

**Results:**

The study material consisted of seven healthy horses and seven horses with symptomatic RAO. All horses were exposed to moldy hay and straw for 48 h to induce clinical exacerbation of RAO. The serum 8-OHdG levels were determined using the ELISA Highly Sensitive 8-OHdG kit. The difference between the levels of 8-OHdG in healthy and RAO-affected horses was significant. The median level of 8-OHdG was 0.044 ng/ml in the healthy controls versus 0.498 ng/ml in RAO horses (*P* = 0.0021).

**Conclusions:**

The results of the study strongly suggest that DNA damage coexists in the course of equine RAO. We therefore propose that future research should aim at the development of new drugs that target pro-inflammatory molecules, since DNA damage appears to be the result of chronic inflammation.

## Findings

Recurrent airway obstruction (RAO) also known as heaves, is an allergic, inflammatory and obstructive airway disease that usually affects older horses and shares many characteristic features with human asthma [1, 2].

Several studies have clearly shown that oxidative stress in the course of equine heaves results from an inflammatory response [3–7]. Moreover, reactive oxygen species (ROS) can generate severe oxidative damage in nucleic acids, such as strand breaks and base oxidations [8]. 8-hydroxy-2-deoxyguanosine (8-OHdG) is one of the best-known oxidation products as it has a high premutagenic potential and is easy to analyze [9, 10]. To date, the level of 8-OHdG has not been reported in the course of equine RAO. Thus, in this study, equine serum levels of 8-OHdG were determined.

All animal experimental procedures in this study were performed with the approval of the 2nd Local Ethics Committee on Animal Experimentation in Wrocław (permission No 1/2012).

Seven Polish Konik horses (five geldings and two mares, median age 8, range 5–13, 6 horses were <10 years old) that had no symptoms of airway disease were used as controls. Seven Polish Konik horses with a history of RAO were included in the study group (four geldings and three mares, median age 9, range 7–14, 4 horses were <10 years old). Samples from these horses have been included in previous publications; therefore some data, i.e. clinical assessment and bronchoalveolar lavage fluid (BALF) cytology are duplicated [11, 12].

All the horses were enrolled in the study based on their medical history, a thorough clinical examination, endoscopy of the airways and the results of a bronchoalveolar lavage fluid cytology. An acute crisis of heaves in RAO-affected horses was induced by placing the horses in a poorly ventilated stable, bedding them on straw and feeding them hay with visible mold growth for 48 h prior to the examination. Airway endoscopy and bronchoalveolar lavage were performed in all the animals using previously described methods [11, 12]. For cytology, sample aliquots were cytospinned at 300×*g* for 10 min (Allegra x-22 cytospin, Beckman Coulter, Brea, USA). Smears were prepared and stained with Wright’s stain. A 400-cell leukocyte differential count (×1000 magnification) was performed wherein epithelial cells were not taken into account [13].

Thawed serum samples were used to determine the level of 8-OHdG by a commercial ELISA kit (Highly Sensitive 8-OHdG Check ELISA; Fukuroi, Shizuoka, Japan). First, the serum samples were passed through 0.5 ml 10 K Amicon Ultra Centrifugal Filters (Merck Millipore, Darmstadt, Germany) to remove any large-molecular-weight substances. Then, 50 µl of a primary monoclonal antibody and 50 µl of a sample/standard were added to the microtiter plate wells, which were pre-coated with the 8-OHdG. The plates were sealed tightly and incubated at 37 °C for 1 h in the dark. After washing with 250 µl of phosphate-buffered saline (PBS), 100 µL of secondary antibody conjugated with horse radish peroxidase was added to each well and incubated at 37 °C in the dark for 1 h. Following a second washing step, 100 µl of the enzyme substrate was added to each well and the reaction was stopped after 15 min by adding 100 µl of 1 M phosphoric acid. Absorbance readings were taken 3 min later with a spectrophotometer operating at 450 nm. The results were expressed as ng/ml serum.

Data are presented as median values and 25th and 75th percentiles. The nonparametric U Mann–Whitney test was used to analyze data using STATISTICA v. 10.0 software (StatSoft, Tulsa, OK, USA). *P* < 0.05 was considered statistically significant.

The results of the BALF analysis, clinical assessment and arterial blood gasometry are shown in Table 1. BALF median recovery in the control horses was 56.6 and 43.6 % in the RAO-affected horses. Statistical analysis demonstrated a significant effect of exposure to moldy hay and straw for 48 h on the percentage of BALF cells in the horses with heaves compared to the controls.

**Table 1:** Results of bronchoalveolar lavage fluid cytology, clinical assessment and blood gas analysis in healthy horses and recurrent airway obstruction (RAO)—affected horses sampled after challenge to moldy hay and straw

	Healthy (n = 7)	RAO-affected (n = 7)
Clinical score^a^	2.0 (2 and 2)	6 (5 and 6)
PaO2 (mmHg)^a^	96 (91 and 108)	91 (85 and 107)
PaCO2 (mmHg)	45 (43 and 46)	45.5 (44 and 50)
BALF neutrophils (%)^a^	5.1 (4.1 and 5.3)	59.8 (51.3 and 64.8)
BALF lymphocytes (%)^a^	41 (38.5 and 45.9)	38.1 (34.8 and 41.1)
BALF macrophages (%)^a^	55.8 (49.8 and 59.1)	32.8 (25.9 and 35.7)
BALF eosinophils (%)^a^	0.4 (0.2 and 0.5)	0 (0 and 0)
BALF mast cells (%)	0.1 (0 and 0.3)	0 (0 and 0)

Values are expressed as median and 25th and 75th percentiles. This table has been published previously [11, 12]

^a^Differences statistically significant (P < 0.05)

**Fig. 1:**
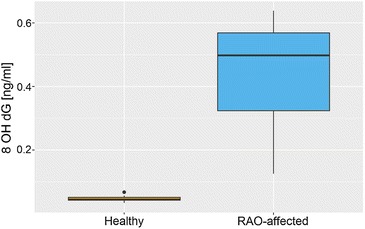
Serum 8 OHdG concentrations in recurrent airway obstruction (RAO)—affected horses and healthy controls determined after challenge to moldy hay and straw. The *horizontal line* represents median value and *whiskers* represent 25th and 75th percentiles

8-hydroxy-2-deoxyguanosine was detected in all serum samples. There was a significant difference in the levels of 8-OHdG between healthy and RAO-affected horses (*P* = 0.0021). The median level of 8-OHdG was 0.044 ng/ml in the healthy controls versus 0.498 ng/ml in RAO horses (Fig. 1).

It has recently been confirmed that equine RAO is accompanied by oxidative stress [14]. The generation of ROS through a number of endogenous mechanisms, including activation of redox-sensitive transcription factors and pro-inflammatory signaling pathways, is critical to the inflammatory response. At the same time, an inadequate antioxidant defense may lead to a chronic and a more pronounced inflammatory response. An exacerbation of RAO is manifested by airway inflammation, including an influx of circulating white blood cells (predominantly non-degenerate neutrophils) into the bronchial lumen [1]. When activated, leukocytes release many pro-inflammatory mediators that amplify the inflammation [15] and leads to cell damage [16].

Another mechanism that may underlie oxidative stress in the course of RAO is hypoxia, which typically develops in horses during disease exacerbation [17, 18]. Equine heaves causes inadequate oxygen supply to the cells and tissues and leads to overexpression of the hypoxia-inducible factor (HIF), which is a major regulator of energy homeostasis and cellular adaption to hypoxia. The expression of the HIF-1 alpha subunit gene (*HIF1A*) is upregulated in myeloid cells of horses with RAO [17]. An in vitro study also showed a significantly increased expression of the *HIF1A* in stimulated peripheral blood mononuclear cells (PBMCs) of RAO-affected horses [18]. *HIF1A* has a central role in the response to hypoxia. A possible cause of the oxidative stress is mediated by the release of the kappa B nuclear factor (NFkB), a transcriptional factor that triggers the inflammatory process [19]. Another explanation of oxidative stress in equine RAO may be an increased anaerobic glycolysis and a loss of electrons from the mitochondrial electron transport chain [20].

DNA and RNA can be damaged by ROS, which are generated in cellular metabolic pathways as a response to exposure to factors, such as UV light or heat. Highly ROS may cause DNA damage, leading to hydroxylation of nucleic acids [21]. Among the multiple products of nucleoside oxidation, 8-hydroxy-2-deoxyguanosine (8-OHdG) and 8-hydroxyguanosine (8-OHG) are two of the most prominent and best-characterized compounds [8–10].

This study confirms an elevated level of serum 8-OHdG in RAO-affected horses, that may be associated with increased level of DNA damage (Fig. 1). To date, no studies assessing the levels of DNA-oxidative damage markers in horses have been reported. However, our results are consistent with other studies where significant increased levels of markers of DNA-oxidative damage in COPD or asthmatic patients were found [22–24].

Despite differences in the results obtained from humans and horses, it is still of concern that RAO-affected horses have almost 10 times higher levels of 8-OHdG compared to healthy horses. It is not clear from the present research how RAO-affected horses developed higher levels of oxidative DNA damage. Recurrent airway obstruction contributes to oxidative damage through an increase in the production of, and exposure to ROS [14]. RAO is associated with an activation of innate immune responses, the promotion of inflammation, and increased vulnerability to latent infection. Activated phagocytes are significant sources of ROS as a part of the cytotoxic host response against invading pathogens [24]. This process may underlie the phenomenon that members of the high-prevalence RAO family show a low degree of intestinal parasitism [25, 26]. However, the ability to induce specific host immune regulatory mechanisms may be partly determined by host genetics. Horses that are genetically susceptible to allergic disease may be more likely to develop allergic responses to nematodes. Based on the above mentioned mechanism, they may be genetically more resistant to parasite infection. Another possibility is that equine RAO affects the repair of damaged DNA. A reduction in the repair process could increase the measured levels of oxidative damage products by allowing their persistence rather than increasing their formation. Future research including simultaneous measurements of innate immune activation, DNA damage and DNA repair, may enhance our knowledge on the pathophysiology of this process.

The demonstration of a shift in the oxidant–antioxidant balance in favor of the former suggests that augmentation of antioxidant defenses by means of therapeutic interventions may be beneficial.

In conclusion, the results strongly suggest that DNA damage coexists in the course of equine RAO. Oxidative stress and consequent DNA damage appear to be the result of chronic inflammation. Therefore, the development of new drugs that target pro-inflammatory molecules or ROS-inducers in the course of equine RAO should be attempted. Moreover, a shift in the oxidant-antioxidant balance in favor of the former suggests that augmentation of antioxidant defenses by means of therapeutic interventions may be beneficial.

## Abbreviations

8-OHdG: 8-hydroxy-2-deoxyguanosine
BALF: bronchoalveolar lavage fluid
HIF: hypoxia-inducible factor
NFkB: kappa B nuclear factor
PaCO_2_: partial pressure of carbon dioxide
PaO_2_: partial pressure of oxygen
PBMCs: peripheral blood mononuclear cells
RAO: recurrent airway obstruction
ROS: reactive oxygen species
